# Effect of rapid thermal annealing on the optoelectronic figures of merit of a vanadium nitride/porous silicon heterojunction photodetector

**DOI:** 10.1039/d6ra09304c

**Published:** 2026-11-11

**Authors:** Sarab T. Kasim, Hasan A. Hadi, Raid A. Ismail, Noor Dhaief Hayif

**Affiliations:** a Educational Rusafa Directorate II Baghdad Iraq; b Department of Physics, College of Education, Mustansiriyah University Baghdad Iraq; c College of Applied Science, University of Technology Baghdad Iraq raidismail@yahoo.com

## Abstract

Rapid thermal annealing (RTA) is an essential step in the fabrication of photovoltaic devices. It considerably improves crystallinity, dopant activation, electrical conductivity, optical efficiency, and contact formation while minimizing unwanted diffusion. These improvements, in turn, facilitate the fabrication of high-performance devices. In this study, vanadium nitride (VN) thin films and porous silicon (PSi) were prepared using reactive DC sputtering and photoelectrochemical etching (PECE) techniques, respectively. The effects of RTA at 1023 K for 35 s on the structural, morphological, optical, and electrical properties of the VN films and the figures of merit properties of VN/PSi/*n*-Si heterojunction photodetectors were systematically studied. X-ray diffraction (XRD) studies confirmed that the crystallinity of the film improved after RTA. The optical energy gap decreased from 3.63 to 3.58 eV after RTA. Scanning electron microscopy (SEM) revealed that the RTA process increased the particle size of the VN film and enhanced grain integration on the porous silicon surface. The fabrication and characterization of the VN/PSi/*n*-Si heterojunction photodetector are demonstrated. The dark *I*–*V* characteristics demonstrated a significant improvement in junction quality, with the ideality factor decreasing from 1.64 to 1.19 and the barrier height increasing from 0.77 to 0.83 V. The annealed photodetector exhibited a photosensitivity of 2300% at −10 V. Its responsivity increased from 2.69 to 3.76 A W^−1^ at 560 nm after RTA. The improved performance of the VN/PSi/*n*-Si heterojunction (HJ) photodetector after RTA open new opportunities for many applications that require enhanced response to low-intensity light accompanied by a significantly high signal-to-noise ratio (SNR), such as high-speed optical communication, environmental sensing and biosensing applications.

## Abstract 2

Rapid thermal annealing (RTA) is an essential step in the fabrication of photovoltaic devices.

## Graphical abstract

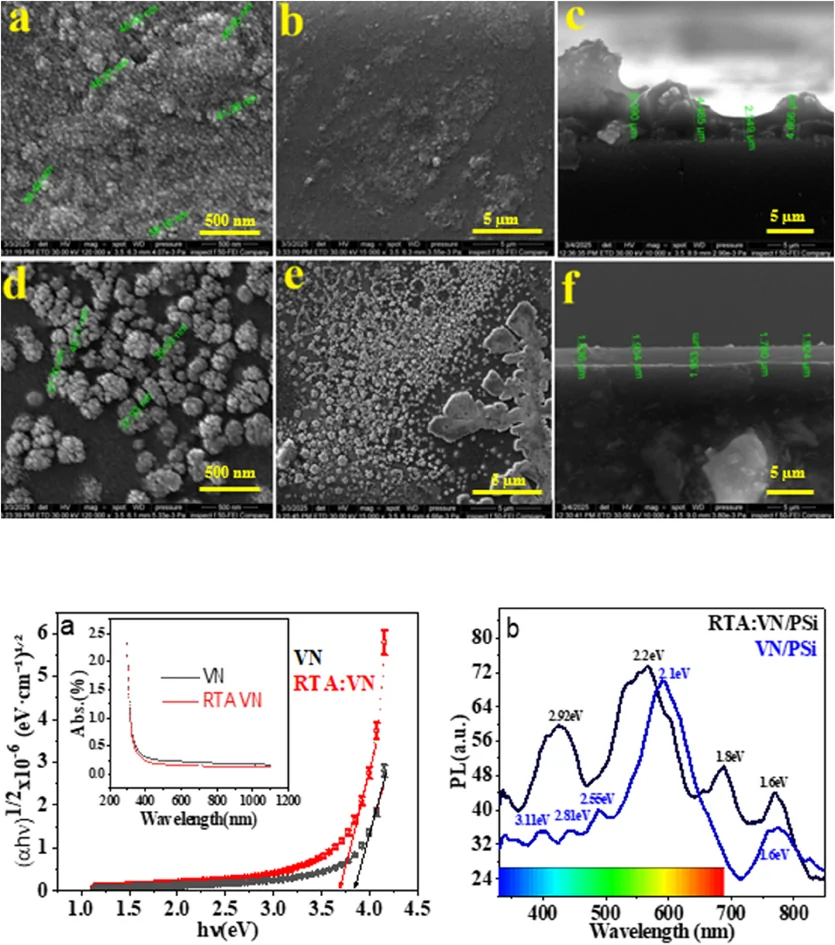

## 1 Introduction

Recently, in many modern applications, the focus has shifted away from materials with a single property. Instead, there is growing interest in materials capable of integrating multiple physical properties to meet the requirements of various applications within a single environment.^1,2^ Transition metal nitrides (TMNs), which exhibit both metallic and semiconductor properties, are therefore well suited for many optoelectronic applications.^3^

In recent decades, the unique physical and chemical properties of vanadium nitride (VN), a transition metal nitride (TMN), have attracted significant scientific attention. Many previous studies have shown that VN has distinctive physical properties, with its electrical properties varying depending on its thickness and nanostructure.^1^ VN is one of the materials that combines mechanical strength, high thermal stability, and good conductivity. Thus, it is a promising material for many electronic, energy storage, supercapacitor, and microelectronic applications.^4,5^

Jia *et al.* found that varying the nitrogen flux during the preparation of VN thin films by DC sputtering could shift the crystallographic orientation from the (200) plane to the (111) plane, significantly affecting the film's hardness and residual stress.^6^ Achour *et al.* investigated the vacuum annealing of VN thin films at different temperatures to optimize their properties and enhance the stability of the oxide layer for potential applications in electrochemical capacitors.^7^ Qiao Luo *et al.* highlighted the significance of vanadium nitride as one of the transition metal nitrides, which are widely used for supercapacitors due to their exceptional physical and chemical properties that are linked to their intrinsic structure.^1^ Pentin *et al.* demonstrated that VN thin films have potential applications in ultra-sensitive detectors, exhibiting an ultra-high transition temperature of approximately 6 K and an electron relaxation time of around 2.6 ns, making them suitable for the fabrication of hot-electron bolometer (HEB) detectors.^5^ Amina *et al.*^8^ demonstrated that vanadium nitride (VN) is a promising material for solar thermal photovoltaic applications owing to its high thermal stability and excellent optical properties. VN exhibited more than 98% absorption in the 300–800-nm range, with maximum absorption at 340 nm, and showed high emission efficiency when integrated with photovoltaic cells, making it a promising candidate for solar energy harvesting.^8^ In a simulation study examining the electronic properties of vanadium nitride (VN), Ammar *et al.*^9^ demonstrated that it exhibits semi-metallic behavior with both zinc-blende and rock salt structures. It acts as a semiconductor for spin-down electrons and a conductor for spin-up electrons. With a direct energy gap of 3.77 eV and a high absorption coefficient, it is a promising material for optoelectronic applications.

The rapid thermal annealing (RTA) technique has been used widely in the short-cycle treatment of semiconductor thin films, where it is used efficiently for microelectronic applications.^7^ Porous silicon (Psi) provides a large surface area owing to its highly nanostructured surface and good ability to absorb light, rendering it a promising substrate for optoelectronic applications. The electrochemical etching technique was used to prepare porous silicon, as it is simple and efficient for producing porous structures. The control of the porous structure can be achieved by varying the preparation conditions. Therefore, it is possible to obtain a basis for heterojunctions and photodetectors.^8^

Heterojunction devices are playing an increasingly important role in various optoelectronic applications, particularly in the design and operation of numerous devices, including photodetectors, solar cells, gas sensors, and many other electronic devices. This is due to their ability to enhance charge carrier transport and convert light energy into an electrical signal. The deposition of a semiconducting thin film of porous silicon to fabricate self-powered, broadband, high-performance photodetectors has been extensively reported,^9,10^ which exhibits a high surface area, excellent light-trapping capability, and good compatibility with silicon technology.^11^ Since the thermal conductivity of porous silicon is strongly dependent on temperature and is primarily governed by its porosity,^12^ controlling the thermal behaviour of such porous structures is essential for optimizing device performance. In this context, thermal annealing is widely employed as an effective post-treatment strategy to modify the structural and optoelectronic characteristics of heterojunction devices. Previous measurements on heterostructures, such as the Ag/Bi_2_Te_3_ heterojunction, have demonstrated that optimized thermal annealing substantially enhances the photodetector performance by promoting crystallinity and reducing trap-assisted recombination.^13^ These structural alterations assist in efficient charge-carrier transport and strengthen optical absorption, which leads to higher photoresponsivity and sensitivity.^13^

Furthermore, similar annealing-induced enhancements have been reported in other heterostructures, where thermal treatment effectively tunes the optical band gap and reduces structural disorder, thereby boosting the overall device efficiency.^14^ Despite the well-documented advantages of annealing in various heterojunctions, its specific impact on the structural and electrical properties of vanadium nitride/porous silicon (VN/PS) heterojunctions has not been thoroughly investigated. Therefore, the present work aims to fill this gap by exploring how thermal annealing influences the performance of VN/PS heterojunctions, building upon the established benefits observed in analogous systems.

Previous studies have reported the performance of vanadium-based photodetectors, which depends on material composition, device structure and detection mechanisms. Bowen Li *et al.* developed vanadium dioxide (VO_2_)-based photothermoelectric detectors, which achieved a photocurrent on/off ratio exceeding 10^5^, a responsivity of approximately 500 A W^−1^, a detectivity of 3.9 × 10^12^ Jones, and rapid rise and decay times of 2 and 5 µs, respectively, under a 1 V bias.^15^ In another study, Shaiju S. S. *et al.* proposed a VO_2_(M)-based metal-semiconductor-metal (MSM) photodetector. The device achieved a photocurrent density of approximately 57 µA cm^−2^ and a response time of 1–2 ms under visible light at 100 mV. At 800 mV and 590 nm, the detectivity reached 3.2 × 10^10^ Jones and the photocurrent density reached 465 µA cm^−2^. The photodetector exhibited a responsivity of approximately 2.54 A W^−1^ and an external quantum efficiency (EQE) of 534%.^16^ Fu-Hai Guo *et al.* presented high-performance Si/VO_2_ nanorod (NR) heterojunction photodetectors based on the photothermoelectric (PTE) effect. Heterojunction engineering has further improved the detectivity to 1.6 × 10^13^ Jones with a rapid rising-edge time of approximately 65 µs.^17^ While these studies demonstrated the photodetection capabilities of VO_2_-based devices, this study aimed to investigate the effect of RTA on the optoelectronic figures of merit of the VN/PSi photodetector. A correlation was established between the surface morphology, optical and electrical properties, and photoresponse of the photodetector following RTA.

## 2 Experimental

The preparation processes involve the following procedures.

### 2.1 Photo-electrochemical etching (PECE)

A PECE technique was used to prepare a porous silicon layer by etching a mirror-like face of an n-type silicon substrate with 12–20 Ω cm electrical resistivity, 1 cm × 1 cm dimensions, 550 µm thickness, and (111) orientation. A platinum electrode and a piece of silicon measuring 1 cm × 1 cm were used as the cathode and anode electrodes, respectively, and were connected with a DC power supply in a beaker containing a 1 : 1 mixture of hydrofluoric acid (HF) and methanol electrolyte. A 40 mW continuous-wave (CW) diode laser with a wavelength of 650 nm was used to illuminate the surface of the mirror silicon face during the etching process. The experimental setup used for electrochemical etching was presented in a previous study.^18^

### 2.2 Reactive DC sputtering (RDCS)

DC magnetron sputtering was employed to deposit vanadium nitride thin films on porous silicon and conventional glass substrates in a nitrogen-containing atmosphere, as described in our previous work.^19^ The target was positioned 11 cm from the substrate holder, which was selected as the optimum distance to achieve the desired film thickness during the 1 h deposition period. Gas ionization was initiated by applying a DC power of 100–120 W to the target to sustain the plasma and achieve a deposition rate of 6–7 nm min^−1^. The total pressure in the vacuum chamber was maintained at approximately 8 × 10^−2^ mbar. The applied voltage was 2.5 kV, while the discharge current ranged from 10 to 15 mA. The partial pressures of argon (Ar) and nitrogen (N_2_) were 6 × 10^−2^ and 2 × 10^−2^ mbar, respectively.

### 2.3 Rapid thermal annealing (RTA)

The rapid thermal annealing process was conducted using an RTA system consisting of a bank of halogen lamps with reflectors, a quartz chamber, temperature control, a K-type thermocouple, a vacuum system, a sample holder, and N_2_ gas flow. The annealing was carried out at an annealing temperature of *T*_A_ = 1023 K and an annealing time of *t*_A_ = 35 s.^20,21^Fig. 1 shows a schematic of the RTA system used for annealing the VN film.

**Fig. 1:**
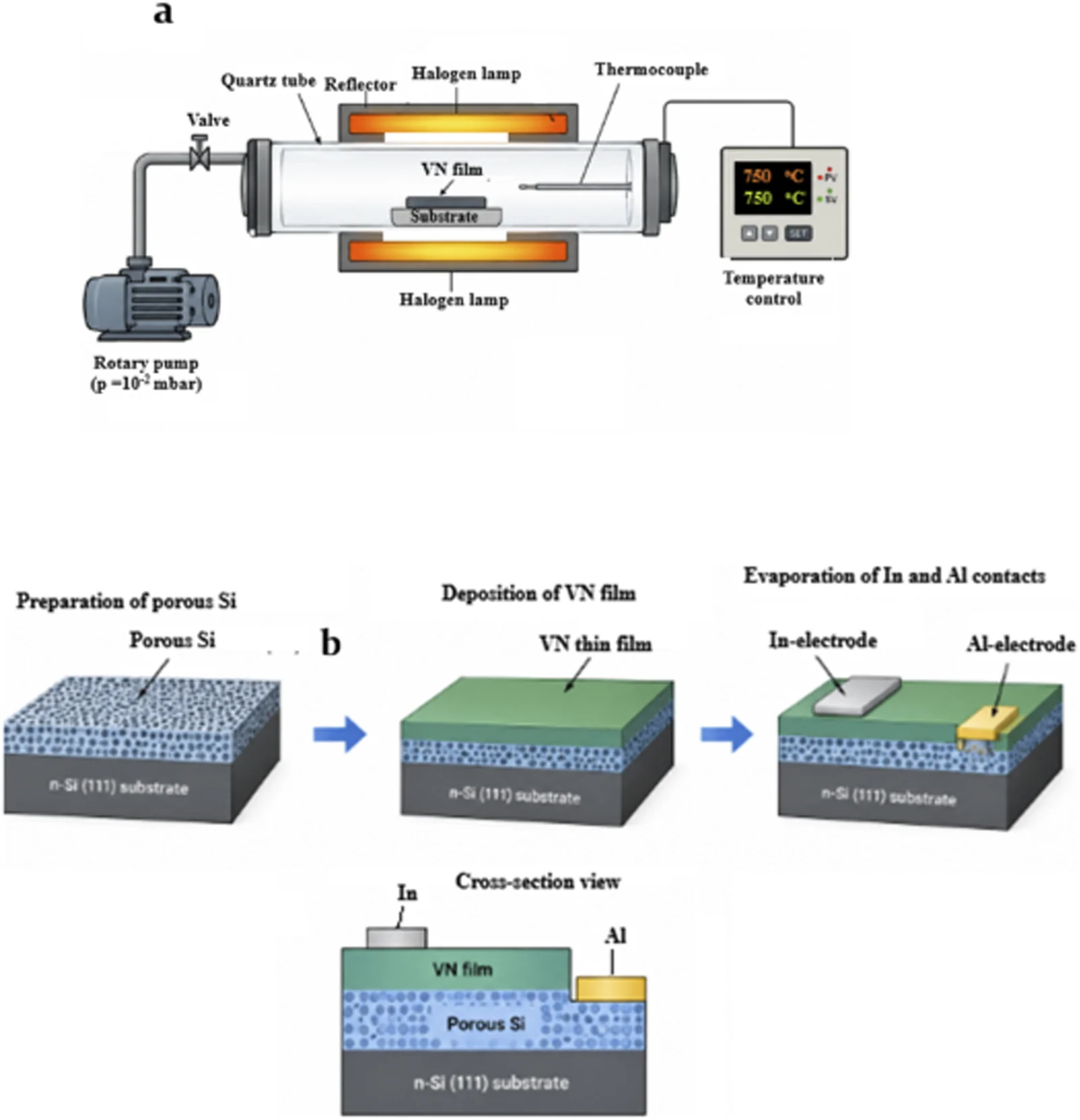
(a) Schematic of (a) RTA system and (b) fabrication steps of the In-VN/PSi-Al photodetector.

### 2.4 Characterization

To investigate the surface morphology of VN films deposited on glass and porous silicon substrates, SEM images were acquired using field emission scanning electron microscopy (FE-SEM, Inspect f 50-FEI business) at an accelerating voltage of 30 kV and a working distance of 6.3 mm, equipped with an Everhart–Thornley detector (ETD). The particle size was determined from the SEM images using the ImageJ software by selecting 15 measurement points to calculate the average particle size. The crystal structure of the VN thin films was characterized using a PANalytical Aeris X-ray diffractometer equipped with an Empyrean Cu LFF HR X-ray tube and a PIXcel1D-Medipix3 detector. Cu Kα radiation (*λ*Kα_1_ = 0.1540598 nm) was used at an operating voltage of 40 kV and a tube current of 7.5 mA. A nickel filter was employed to suppress the Kβ radiation. The measurements were performed in the continuous scanning mode, with a counting time of 6.12 s. The sample was rotated during the measurement, with a revolution time of 2 s per rotation. The scan range was 2*θ* = 7.01°–99.99°, as specified in the measurement file. A Raman spectrophotometer (Bruker SENTERRA, Germany) was used to identify the vibrational modes with laser excitation wavelengths of 532 and 580 nm. Raman spectroscopy measurements were performed using a Sunshine spectrometer (software version V2_86; serial number 220220190928028605320700). The integration time was recorded as 10 000 000 µs (equivalent to 10 s) with an average count of 1. The spectra were acquired in the original Raman-shift mode, with 1751 data points. The *x*-axis represents the Raman shift in cm^−1^, while the *y*-axis represents the intensity in counts. The instrument software also recorded a smoothness setting of 536 and a reserved parameter value of 250. The photoluminescence (PL) spectra of the VN/PSi/*n*-Si heterojunction were recorded using a FluoroMate FS-2 spectrometer. A 150 W continuous wave xenon-arc lamp was used as the excitation source, and a photomultiplier tube (PMT) was employed for detecting both the excitation and emission spectra, with a photodiode serving as a reference detector. The measurements were performed over the wavelength range of 190–900 nm. To ensure high spectral resolution, the instrument was operated with a minimum resolution of 0.5 nm, a wavelength accuracy of ±0.5 nm, and a wavelength reproducibility of ±0.2 nm. The minimum data interval was set to 0.1 nm. The monochromator was equipped with a 1200-groove per mm grating, blazed at 250 nm for excitation and 400 nm for emission. The instrument demonstrated a sensitivity (S/N ratio) greater than 1000 : 1 (peak-to-peak), evaluated using a water Raman signal with a 10 nm spectral bandwidth. All measurements were conducted in the continuous scanning mode at a scan speed ranging from 1 to 6000 nm min^−1^. The optical absorption spectra of VN thin films deposited on glass substrates, before and after rapid thermal annealing (RTA), were recorded using a JASCO V-670 dual-beam UV-Vis-NIR spectrophotometer. The measurements were performed over the wavelength range of 200–1000 nm, with a scan step of 1 nm and a scan filter setting of 10. Dark/photo-current–voltage (*I*–*V*) characteristics were examined using a DC digital power supply (YAXUN PS-1502DD), a multimeter (Pros Kit MT-1132), and a 150-W halogen light source. The measurements were performed for the Without RTA and With RTA samples in the forward direction over the voltage range from 0 to 10 V and in the reverse direction over the voltage range from −10 to 0 V. The photodetector characteristics were obtained using a digital Mini-Chrome monochromator (DMC1-03 from Optometrics Corporation, USA; 300 nm to 800 nm) and a halogen spectral lamp (PHYWE, Germany). A laser power meter, LP1 (Sanwa, Japan), was employed for power calibration. The responsivity *R*_*λ*_ of the photodetector was evaluated using several optical filters covering a wavelength range from 400 to 1000 nm. The active area of the fabricated device is 0.2826 cm^2^, and the In and Al electrodes were configured as two-point contacts.

## 3 Results and discussion

Fig. 2(a–f) shows the SEM images of the VN films deposited on the glass substrate before and after RTA. Fig. 2(a and b) shows that the morphology of the film consists of aggregated, multifaceted granules that tend to form regular and homogeneous spherical shapes, and after RTA, they become more distinct and regular. SEM analysis revealed that the average particle size before RTA was approximately 41.55 ± 3.56 nm, demonstrating a uniform and narrow size distribution. After RTA, as shown in Fig. 2(d–f), the average particle size increased to 46.8 ± 9.7 nm. These results can be attributed to the atomic rearrangement within the material during the rapid and short-cycle thermal treatment, leading to a reduction in irregular agglomerates and voids. This process promotes crystalline growth. The increase in particle size by 5.25 nm, along with the increased standard deviation, indicates that some grains grew faster than others, resulting in a non-uniform size distribution. The increase growth in the particle size indicates that the film's crystallinity has improved. Fig. 2(c and f) shows the SEM images of the VN film's cross-section before and after RTA, respectively. It is obvious that the thickness of the VN film after RTA is uniform over the entire film.

**Fig. 2:**
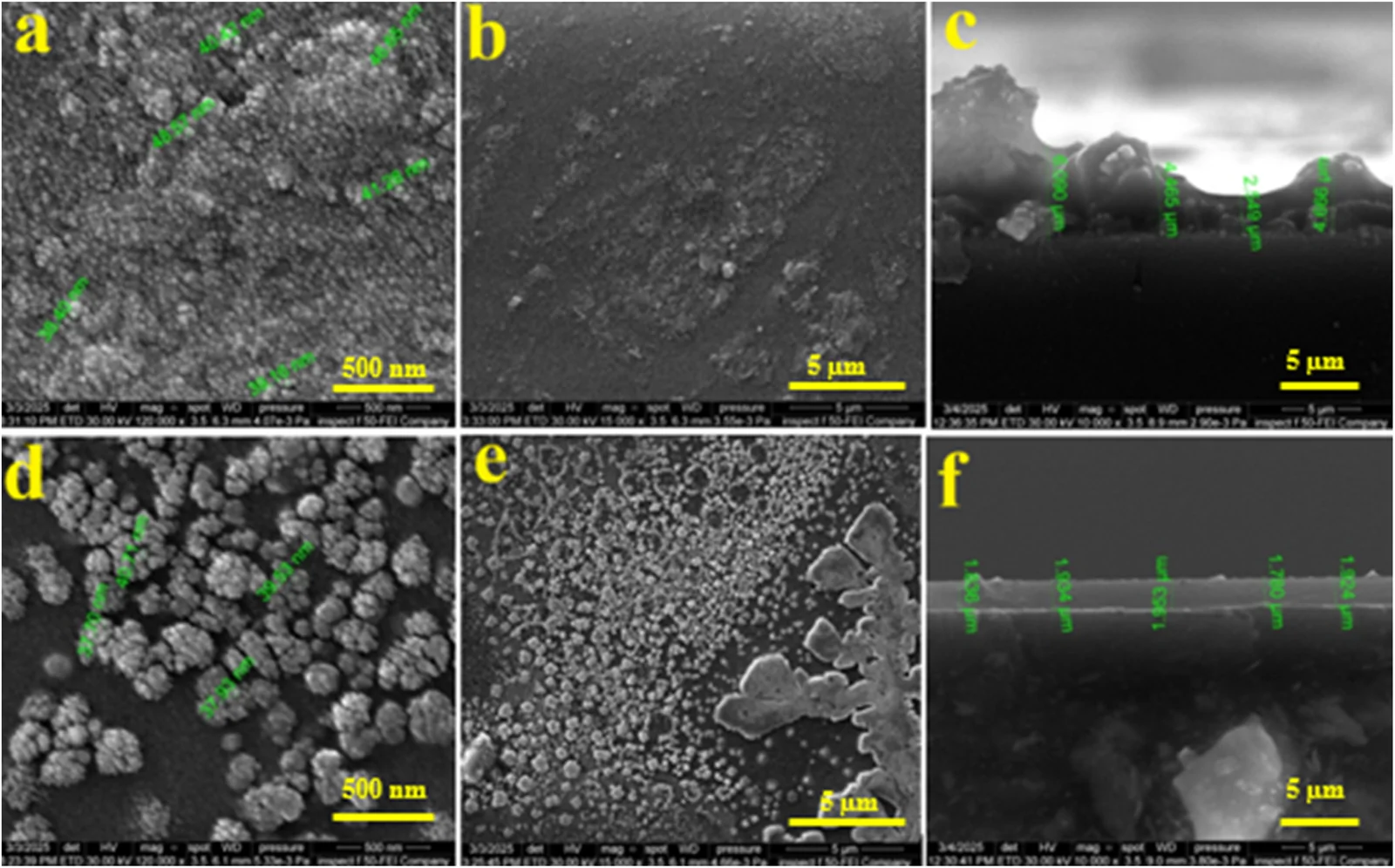
Top-view and cross-sectional FESEM images (at two magnifications) of the VN film/glass substrate before and after the RTA process: (a and b) top-view images at 500 nm and 5 µm and (c) cross-sectional view before RTA; (d) and (e) top-view images at 500 nm and 5 µm and (f) cross-sectional SEM image after RTA treatment.

Fig. 3(a and b) shows the scanning electron microscopy (SEM) images of vanadium nitride films deposited on porous silicon substrates before RTA. The prepared vanadium nitride films exhibited a pronounced non-uniformity in shape yet consistent distribution of the deposited particles on the porous silicon substrate. This behaviour is attributed to the porous nature of the surface, which plays a significant role in the formation and distribution of the particles. The irregularity and lack of homogeneity in the grain shape on the porous silicon layer before RTA are due to the porous surface nature, which greatly contributes to the formation and distribution of VN grains. As shown in Fig. 3(d and e), the grains become larger and their distribution becomes broader and more integrated after RTA compared to those on glass substrates. From the SEM analysis, the average particle size of the VN thin film before rapid thermal annealing (RTA) was calculated to be 20.51 ± 4.48 nm. After RTA, the average particle size increased by about 18 nm, reaching 38.42 ± 5.34 nm. The particle size increase observed in the film deposited on the PSi substrates is approximately 13 nm greater than that observed for the film deposited on the glass substrate. This difference can be attributed to the porous nature of the silicon layer, which provides a large surface area that allows for rapid atomic diffusion and faster growth while maintaining a heterogeneous particle size distribution. It significantly promotes crystal growth, which agrees with previous studies on thin-film heat treatment.^22^ Furthermore, variations in growth rates are the common causes of increased size dispersion after RTA processing.

**Fig. 3:**
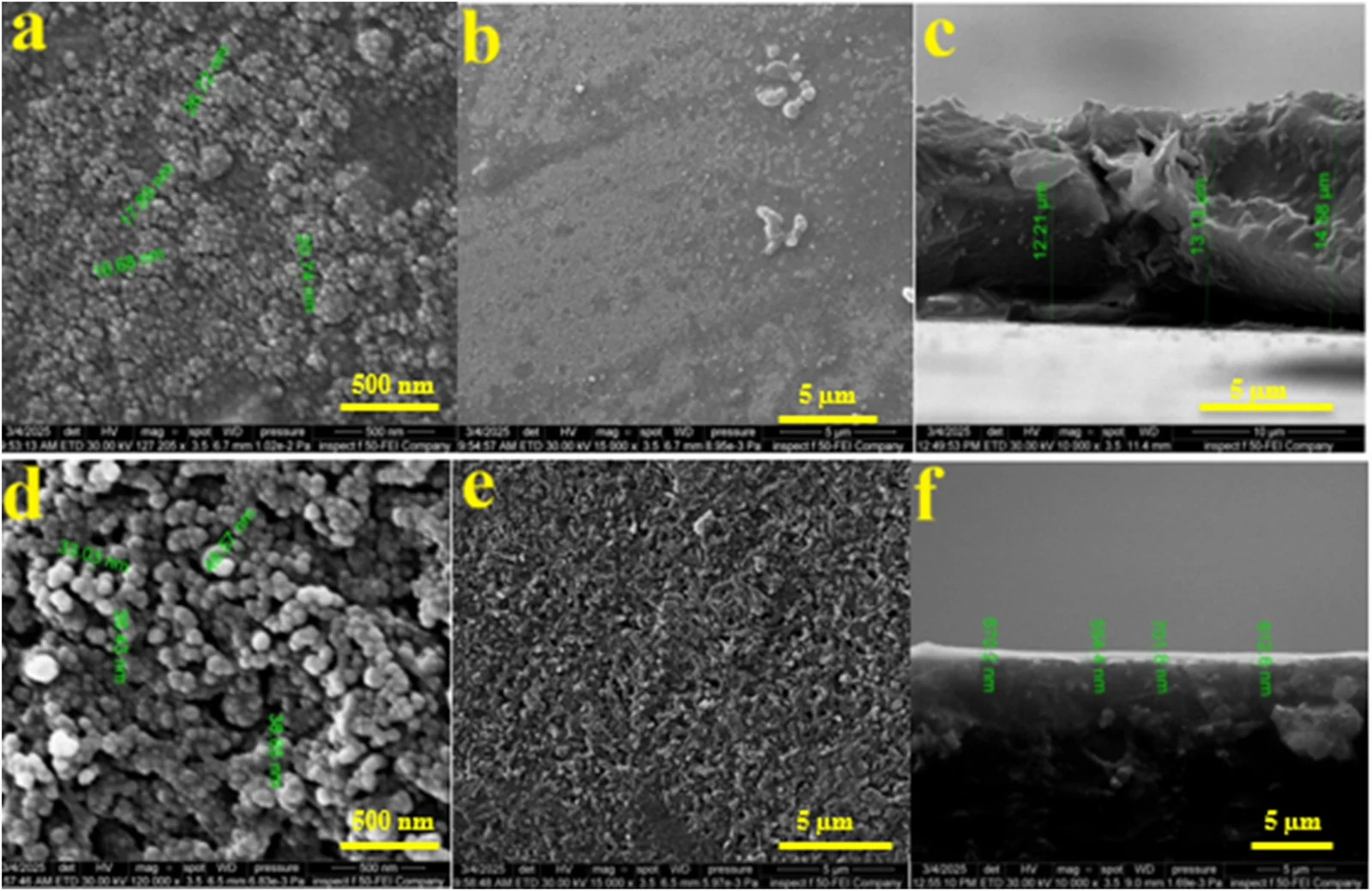
Top-view and cross-sectional FESEM images (at two magnifications) of the VN film/porous silicon before and after the RTA process: (a) and (b) top-view images at 500 nm and 5 µm and (c) cross-sectional view before RTA; (d) and (e) top-view images at 500 nm and 5 µm and (f) cross-sectional image after RTA treatment.

To investigate the structure of the vanadium nitride films prepared on porous silicon substrates before and after rapid thermal annealing (RTA), XRD was performed, and the obtained diffraction patterns were confined to the 2*θ* range of 0°–80°, as shown in Fig. 4(a). The XRD patterns of the VN films before and after RTA show diffraction peaks at 2*θ* = 37.56°, 63°, and 75°, which correspond to the (111), (220) and (311) planes, respectively. These peaks belong to crystalline vanadium nitride with a cubic phase according to JCPDS # 35-0768. A strong peak pattern is also observed at a diffraction angle of 68.79°, corresponding to the (400) plane, which belongs to porous silicon. The lattice constant of the VN film before and after annealing was found to be 4.11 and 4.1 Å, respectively, which is close to that of bulk VN (4.13 Å). The crystallite size of the VN film was estimated using the Debye–Scherrer equation:^23^1
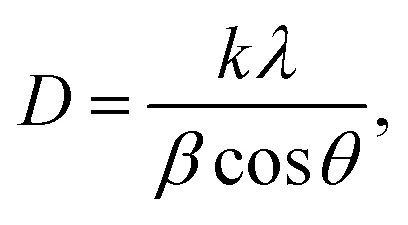
where *D* is the crystallite size (nm), the *K* factor is ≈0.9, *λ* is the wavelength of X-ray, *β* is the FWHM (radians), and *θ* is the Bragg angle (degree).

**Fig. 4:**
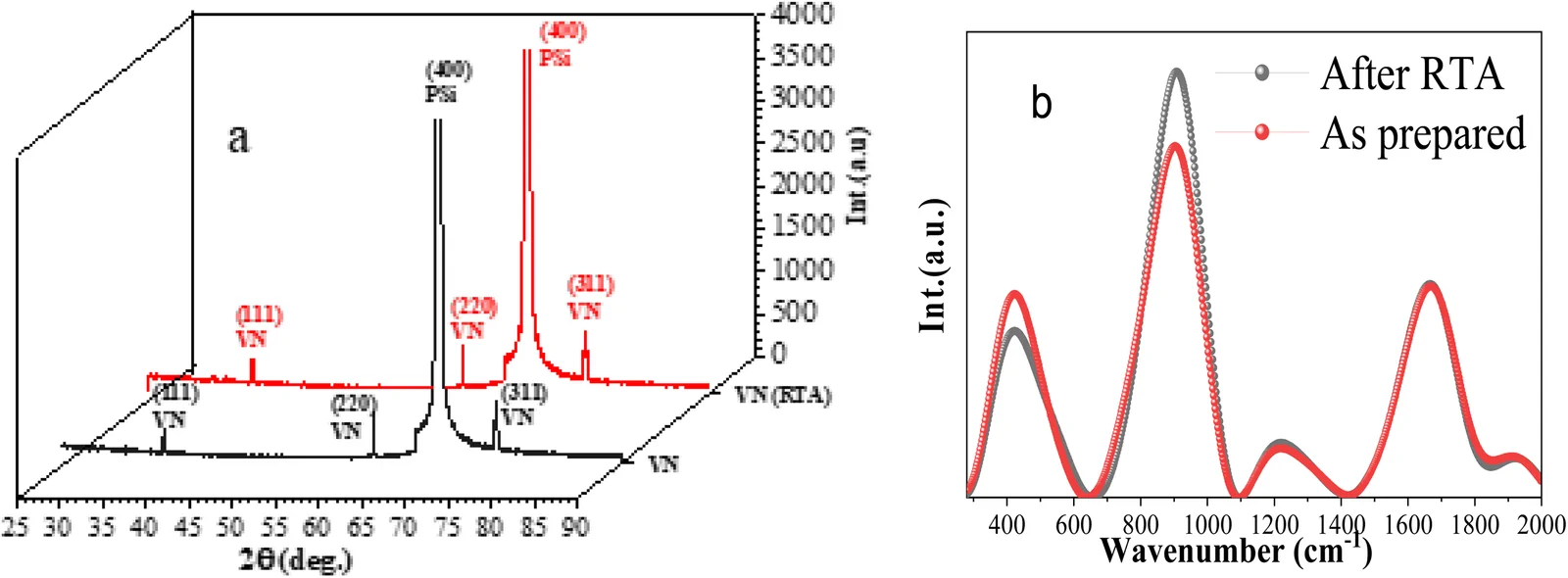
(a) X-ray diffraction patterns of vanadium nitride on porous silicon without (black line) and with (red line) RTA treatment. (b) Raman spectra of the grown VN without RTA (red line) and VN with RTA treatment (black line).

After RTA, the results in Table 1 show a decrease in the full width at half maximum (FWHM), improved peak intensity, and an increase in the crystal size by 43 nm of peaks in the XRD pattern. It can be observed that there is a substantial improvement in crystallinity and structural order, indicating effective recrystallisation without any adverse effect after the RTA process on the lattice constant or causing a phase shift, where there was a significant increase in the average crystallite size of 48% and 112% toward the (220) plane, and the peak narrowing of the FWHM was 52.8% in the (220) plane.

**Table 1:** XRD properties and crystallographic parameters of the VN film deposited on PSi

VN/PSi peaks	Plane	2*θ* (deg.)	FWHM (deg.)	*D* (nm)	Avg. *D* (nm)	*ε*	*Δ*
VN/PSi	210	32.56	0.0755	109.6	90.9	0.000316222	8.32221 × 10^−13^
VN/PSi	220	61.32	0.1279	72.20		0.000480056	1.91796 × 10^−14^

**After RTA**							
VN/PSi	210	32.49	0.0714	115.8	134.3	0.000299103	7.44554 × 10^−13^
VN/PSi	220	61.27	0.0604	152.8		0.000226762	4.27953 × 10^−13^

**PSi/*n*-Si**							
PSi/Si	100	68.79	0.0736	130.8	123.65	0.000264993	5.8442 × 10^−13^
PSi/Si	100	68.72	0.0826	116.5		0.000297522	7.36703 × 10^−13^

The structural analysis section has been expanded to include the dislocation density calculated as follows:

2 *nλ* = 2*d* sin *θ*,

where *n* denotes the diffraction order, *λ* is the wavelength of the X-ray beam, *d* indicates the spacing between the crystal planes, and *θ* is the Bragg diffraction angle.

The lattice strain (*ε*) and dislocation density (*δ*) were calculated as follows:3
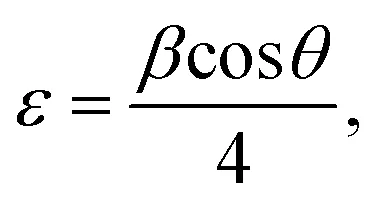
4
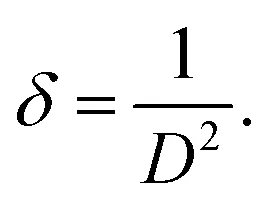


This approach is supported by the results recently reported in the literature: Giri *et al.*^24^ and Priyadarshini *et al.*^25^ demonstrated that dislocation density and microstrain are key indicators of structural quality and directly influence the optoelectronic properties of chalcogenide and heterostructure systems. Mahapatra *et al.*^26^ further confirmed that annealing-induced changes in crystallinity and lattice disorder strongly affect the structural quality and photodetector performance of Bi_2_S_3_ films.

This behavior is common for thin films prepared by physical methods, where atom rearrangement occurs within the crystal lattice, crystal defects are reduced, and crystal growth is promoted. The SEM analysis also showed that the improvement in results is consistent with the XRD results.

Fig. 4(b) shows the Raman spectra of vanadium nitride before and after the RTA process. Four peaks are shown in the low- and high-frequency regions at 420, 902,1282, and 1665 cm^−1^. Two main phenomena can be identified in the spectrum: the first one shows increased intensity and the other one shows a peak shift. The peak at 420 cm^−1^ is assigned to the bending vibrations, and the peak emerging at 902 cm^−1^ is ascribed to the stretching vibrations of vanadium oxide species; VN-related contributions may also be observed. The peak appearing at 1282 cm^−1^ may be attributed to the surface-related species, defects, or carbon-containing residues, and the peak observed at 1665 cm^−1^ is assigned to carbonaceous species or surface contamination, depending on the synthesis conditions. We attribute the increase in the peak intensity after RTA to grain growth, which features increasingly sharp peaks in the XRD patterns, especially toward the (220) plane compared with that toward the 210 plane due to the reduction in FWHM, as shown in Table 1. The peaks appear clearly at 400 and 904 cm^−1^, which are attributed to the vibration modes of VN.^27^

The optical absorption spectra of the VN film before and after RTA are shown in the inset of Fig. 5. The absorption decreases as the wavelength increases within the range of 200 to 1000 nm. The optical forbidden energy *E*_g_ of the sample was calculated by plotting the variation in (*αhν*)^*r*^ with photon energy (*hν*), where *r* is 0.5 for the allowed direct transitions,^28^ as follows:

5 *αhν* = *A*(*hν* − *E*_g_)^*r*^,

where *α* is the absorption coefficient and *A* is a constant that depends on the material.

**Fig. 5:**
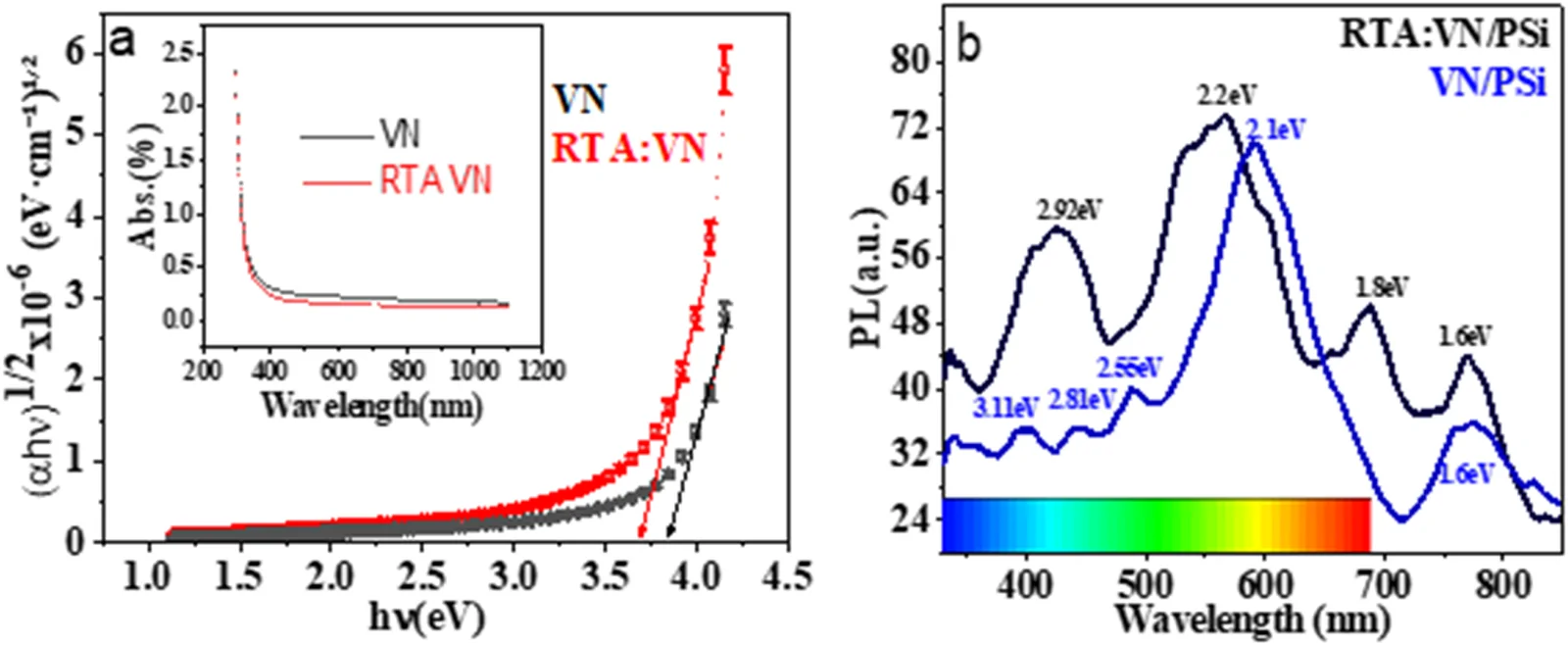
(a) Optical energy gap and spectral absorbance of the vanadium nitride films before and after RTA. Inset shows the optical absorption plot. (b) Photoluminescence spectrum of the VN/PSi/*n*-Si heterojunction.

The linear fit can be performed multiple times over the linear region of the Tauc plot, and the standard deviation of the intercept can be calculated as follows:

6 (*αhν*)^1/2^ = *Ahv* − *AE*_g_,

7 *y* = *mx* + *b*,

Let (*αhν*)^1/2^, *x* = *hν*, *m* = *A* and *b* = −*AE*_g_ than *A* = Slope8
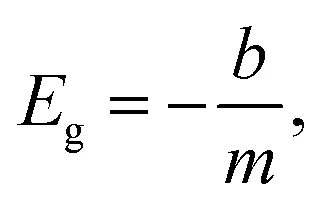


The value of *A* was determined to be 7.592 × 10^−6^.

By extending the linear part of the plot to the photon energy axis (*αhν*)^2^ = 0, the optical energy *E*_g_ can be estimated, as shown in Fig. 5(a). As shown in this figure, the optical energy gap decreased from 3.63 to 3.58 eV after RTA. This finding can be attributed to the fact that, after RTA, the density of local states and the structural disorder are reduced, and this, in turn, modifies the electronic band structure of the VN film. Additionally, RTA can cause the redistribution of nitrogen, which means that the annealing affects the optical transition. On the other hand, the increase in particle size after RTA results in a decrease in the optical energy gap of the film. The photoluminescence (PL) spectra of the VN/PSi/*n*-Si heterojunction before RTA (blue line) and after RTA (black line) are illustrated in Fig. 5(b), where the spectra show various peaks that appear at different energies in the visible region of the spectrum. The observed peaks can be explained according to their spectral regions. The peaks near the UV region are attributed to transitions close to the optical energy gap, such as 3.11 eV and 2.92 eV. The difference between the PL peak energy and that calculated from the absorption edge energy in the UV-visible spectra was 0.69 and 0.78 eV, which is attributed to the stock shift (Δ*E*).^29^ Additionally, the surface defects in porous silicon contribute to this phenomenon, especially for 2.81 eV and 2.55 eV. The optical gap energy level defects, such as surface defects, interfaces (VN/PSi and PSi/*n*-Si), and voids (VN thin film and PSi), are major contributors to the appearance of PL peaks in the visible region, especially in semiconductor nanostructures.^30–32^

These defects act as centres for the radiative recombination of carriers. Other peak energies include those of the near-green region, where peak energies are in the range of 2.2 and 2.1 eV, and those of the near-red region, ranging from 1.6 to 1.8 eV.^30,32–34^Table 2 summarizes the energy differences resulting from defects and their types, classified according to eqn (9):^35^

9 Δ*E* = *E*_abs_ − *E*_PL_.

*E*_abs_ is the band gap from absorption, *E*_PL_ is the photoluminescence peak energy, and Δ*E* or (*E*_def_) is the difference between the peak absorption energy and the PL peak energy, which is attributed to the defects associated with energy levels within the optical gap and the relaxation processes of charge carriers. The energy level defects were calculated before and after treatment, as shown in Table 2, which illustrates the defect energy shift and redistribution in the deep levels inside the energy gap due to the RTA process modification and defect distribution.

**Table 2:** Extracted defect-related energy levels from photoluminescence (PL) spectra before and after the VN/PSi/*n*-Si heterojunction annealing using the RTA technique

PL peaks	Initial		RTA		Defect level analysis	Ref.
	*E*_PL_ eV	*E*_def_ Ev	*E*_PL_ eV	*E*_def_ eV		
Band edge	3.11	0.69	2.92	0.87	Stokes shift, near-band-edge transition 0.69–0.78 eV	29
Peak 2	2.91	0.89	2.2	1.5	Shallow defects and donor states 0.89–1 eV	35
Peak 3	2.81	0.99	1.8	1.9		
Peak 4	2.55	1.25	1.6	2.1	Defect recombination and donor–acceptor transition 1–1.2e V	30–32
Peak 5	2.1	1.7			Si–O bonding, surface state and Si/SiO (porous silicon) 1.6–2.2 eV, deep defect and deep trap states	30 and 32–34
Peak 6	1.6	2.2				

Fig. 6(a) shows V, N, Si, and O as the main elements, with minor traces of C, Mg, Al, K, and Mn, and Fig. 6(b) reveals that the oxygen signal decreases, while the V and N peaks increase in intensity, showing reduced oxidation and improved VN stoichiometry.

**Fig. 6:**
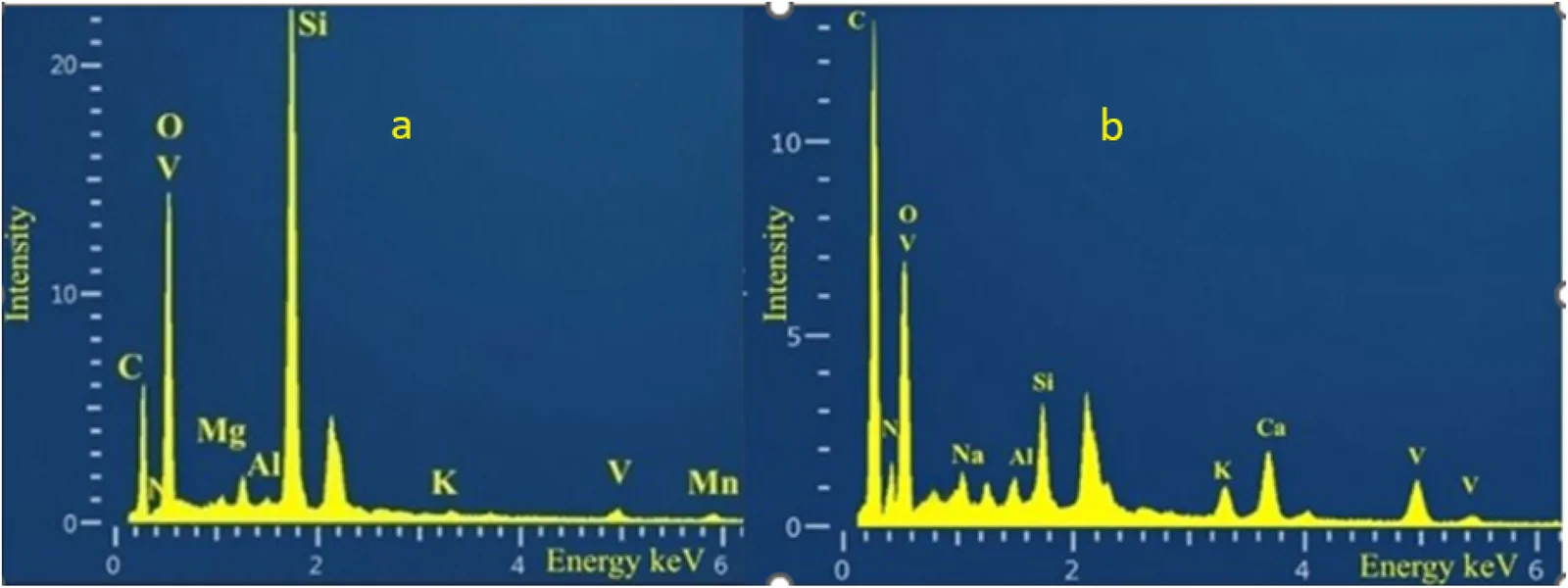
EDX spectra of the vanadium nitride films (a) before and (b) after RTA.

To determine the main parameters (rectification ratio (*R*_r_), saturation current (*I*_o_), barrier voltage height (*∅*_b_), and ideality factor (*n*), which influence the electrical behaviour and performance of the junction in optoelectronic applications), the dark current–voltage (*I*–*V*) characteristics under the forward and reverse bias of the heterojunction VN/PSi/*n*-Si were studied. Fig. 7(a) illustrates the semi-logarithmic plot of the dark current *versus* voltage, and the dark *I*–*V* characteristics representing the behaviour of the VN/PSi heterojunction under forward and reverse bias before and after RTA treatment are shown in the inset of Fig. 7(a). The curves show the formation of an asymmetric heterojunction due to the different current behaviors, with an exponential increase in current under forward bias (a narrow depletion layer) and a relatively small current under reverse bias (a wide depletion layer).This indicates that the device functions as a diode junction.

**Fig. 7:**
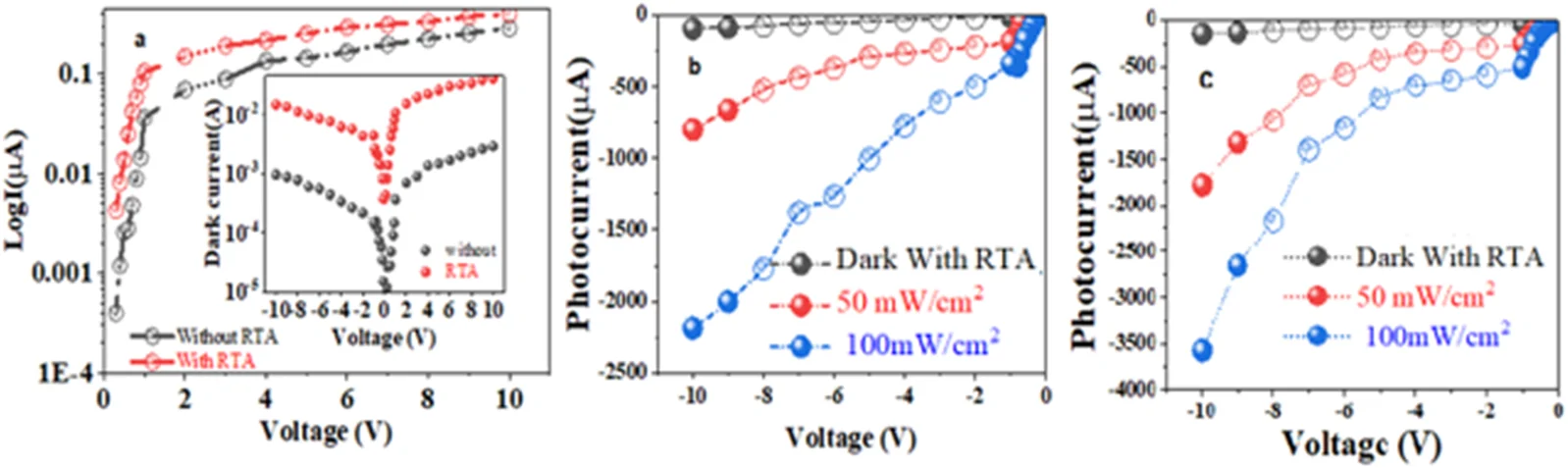
(a) Semi-logarithmic plot of the dark current *versus* voltage and dark *I*–*V* characteristics before and after RTA (inset). (b and c) Photocurrent–voltage characteristics of VN/PSi/*n*-Si without and with RTA under 50 and 100 mW cm^−2^ illumination intensities, respectively.

Based on the thermionic emission theoretical model, the diode equation (Shockley) was used in metal/semiconductor junctions and heterojunction photodetectors as follows:^36^10
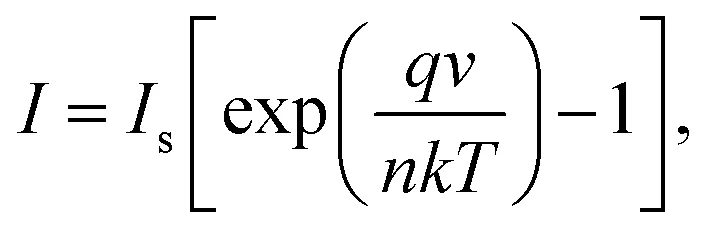
where *I*_s_is the reverse saturation current, *q* is the electronic charge, *k* is the Boltzmann constant, *T* is the absolute temperature in Kelvin, and *V* is the applied voltage. The reverse saturation current can be calculated from the following relation:11
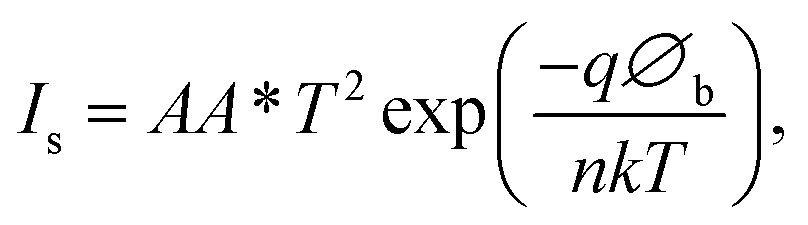
where *∅*_b_ is the Schottky barrier height, *A* is an effective area, and A* is the modified Richardson constant.

As demonstrated in Fig. 7(a), the reverse saturation current was estimated by extrapolating the linear part of the ln(*I*)–*V* curve that intersects the voltage axis at *v* = zero. The barrier height voltage was calculated using the following relation:12
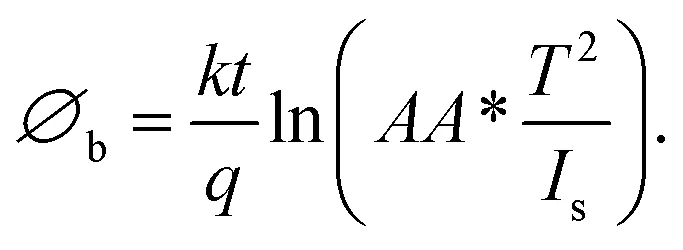


The ideal factor was calculated using the following equation:13
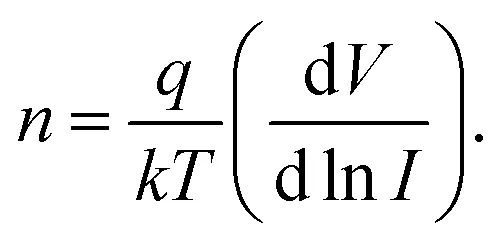


Table 3 summarized the results of the quantitative VN/PSi/*n*-Si heterojunction parameters before and after RTA. A decrease in the barrier height voltage is linked to RTA processing through grain growth, where the total electrical resistance is reduced, and there is a substantial enhancement in electrical conductivity. Concurrently, rapid thermal annealing facilitates the redistribution of thermally generated charge carriers across the VN/PSi/*n*-Si heterojunction, which may contribute to the observed increase in saturation current after RTA. An ideality factor of 1.64 for the VN/PSi/*n*-Si junction before RTA suggests a relatively high density of interface trap states, with recombination through these states contributing to the non-ideal diode behaviour. In contrast, a reduced ideality factor of 1.19 after RTA suggests a reduction in defect-related recombination and a possible passivation of defect states and dangling bonds following the annealing treatment.

**Table 3:** Junction characteristics of the photodetector determined from the dark *I*–*V* measurements before and after RTA

Sample	*I*_s_ (A)	*φ*_b_ (V)	*n*
VN/PSi/*n*-Si	10^−6^	0.77	1.64
VN/PSi/*n*-Si (RTA)	10^−7^	0.83	1.19

The photocurrent–voltage (*I*–*V*) characteristics under illumination are important parameters for evaluating the photodetector performance, as they provide direct information about the conversion of incident light into electrical current. Fig. 7(b) and (c) shows the variation in photocurrent as a function of reverse-bias voltage when the VN/PSi/*n*-Si heterojunction was exposed to halogen illumination at the intensities of 50 and 100 mW cm^−2^, respectively, before and after RTA. The results demonstrate that the VN/PSi/*n*-Si heterojunction exhibits a clear photoresponse under reverse-bias conditions both before and after RTA. The photocurrent increases with the magnitude of the reverse-bias voltage, which can be attributed to the enhanced electric field across the VN/PSi/*n*-Si heterojunction. This electric field facilitates the separation and transport of photogenerated electron–hole pairs toward the respective electrodes, thereby reducing the probability of carrier recombination. It can also be observed that the noise level is relatively low when the dark current approaches zero. Rectification behaviour is clearly observed in the photodiode, as evidenced by the photocurrent–voltage characteristics, which are governed by the potential barrier at the VN/PSi/*n*-Si interface. The switching behaviour under illumination improves with the increasing light intensity. As shown in Fig. 7(c), a significant increase in photocurrent and photosensitivity is observed after RTA. The photosensitivity (*S*) can be determined using the following relationship:^29^14
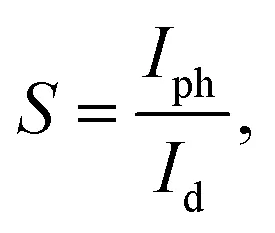

15 where *I*_ph_ = *I*_light_ − *I*_d_.

As shown in Fig. 7(c), a significant increase in photocurrent and photosensitivity is observed after RTA. Photosensitivity (*S*%) is defined as the ratio of the difference between the photocurrent and dark current to the dark current. Under an illumination intensity of 100 mW cm^−2^, the photocurrent increased from 2.2 to 3.6 mA at the maximum reverse-bias voltage, corresponding to a 63.6% increase in the photocurrent. This enhancement can be attributed to the structural and morphological changes induced by RTA, which provides thermal energy that promotes recrystallisation and grain growth in the VN thin film. Furthermore, RTA may reduce the density of trapping and recombination centres for photogenerated carriers, thereby facilitating more efficient carrier transport and collection.

Fig. 8(a) shows the spectral responsivity plot of the VN\PSi photodetector. The responsivity (*R*_*λ*_) of the photodetector was calculated using the following equation:^37^16
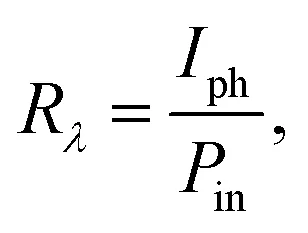
where *P*_in_ is the incident power of the light (W). From Fig. 8(a), it can be confirmed that the peak response of the photodetector was 480 nm before RTA and became 560 nm after RTA. This result can be ascribed to the decrease in the optical energy gap after RTA, leading to a red-shift. These peaks of response are well matched with the absorption edge of the VN films. The responsivity of the photodetector increases after RTA due to the improvement in photocurrent and the decrease in structural defects. The small peak response at 920 nm is due to the absorption edge of the silicon substrate.

**Fig. 8:**
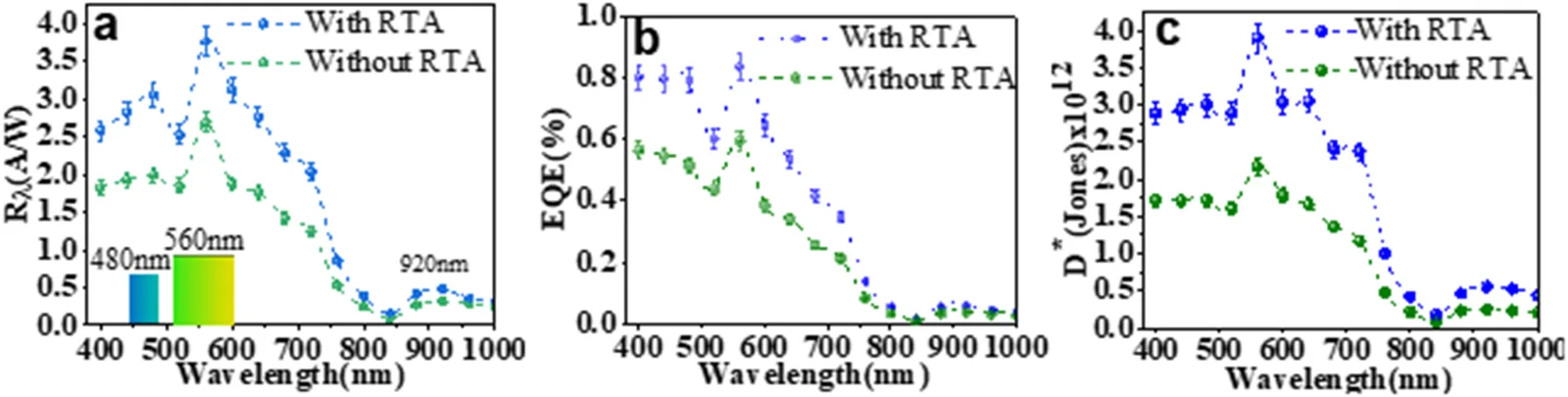
Figures of merit of the VN/PSi/*n*-Si heterojunction photodetector before and after the RTA process: (a) spectral responsivity, (b) external quantum efficiency, and (c) specific detectivity.

The external quantum efficiency of the photodetector can be estimated using the following relationship:^19^17
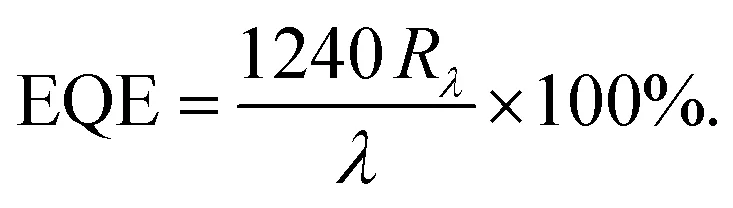
Here, this equation shows the dependence of the EQE on the responsivity and wavelength.

The EQE exhibits a maximum response in the visible region, with a pronounced enhancement after rapid thermal annealing. At 560 nm, the responsivity increases from 2.69 to 3.76 A W^−1^ following RTA, corresponding to an increase in EQE from approximately 595.5% to 832.9%, as shown in Fig. 8(b). This enhancement indicates improved photocarrier generation, separation, and collection following the thermal treatment. It should be noted that the observed EQE maximum at 560 nm cannot be attributed solely to the band-to-band absorption in VN. The EQE values exceeding 100% can be attributed to the internal photoconductive gain, which may occur when the lifetime of photogenerated carriers is longer than their transit time through the device. The (EQE) exceeding 100% can be associated with photoconductive internal gain, which is commonly described by the relationship 
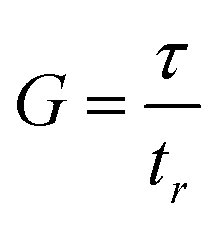
, where *τ* is the carrier life time and *t*_r_ is the transit time. A calculated *τ* is longer than *t*_r_, photogenerated carriers can facilitate the collection of multiple charge carriers through the external circuit before recombination, leading to an apparent EQE exceeding 100%. This lifetime-to-transit-time relationship has been widely discussed in the literature on photoconductive and photogating-based photodetectors.^38,39^ In this study, the maximum EQE increases from 595.5% for VN/PSi to 832.9% for RTA-treated VN/PSi at 560 nm, suggesting an enhanced photoresponse following annealing. This improvement may be related to the changes in carrier transport and defect-associated trapping and recombination processes.^38,39^

The optical band gap of the VN film is approximately 3.58 eV, corresponding to an absorption-edge wavelength of about 346 nm, whereas the photon energy at 560 nm is only about 2.21 eV. Therefore, the visible and near-infrared photoresponse is more reasonably associated with the combined contributions of the VN layer, porous silicon, the underlying silicon substrate, interface states, and defect-related or sub-bandgap absorption processes. The enhanced response after RTA may result from the improved interfacial properties and more efficient photocarrier transport and collection, together with the modifications of the defect states within the heterojunction. At longer wavelengths, the decrease in the EQE is consistent with the lower photon energy and the wavelength-dependent absorption and carrier-collection processes of the heterostructure.^40,41^ Therefore, the spectral dependence of the EQE should be interpreted in terms of the combined optical and electronic properties of the VN/PSi/*n*-Si heterojunction rather than solely in terms of the optical band gap of VN. One of the most important key metrics for measuring a detector's actual performance is its ability to detect weak signals and thus its signal-to-noise ratio (SNR) within a 1-Hz noise bandwidth using a 1 watt light source. The specific detectivity of the VN/PSi/*n*-Si HJ photodetector before and after RTA is shown in Fig. 8(c). Since the specific detectivity plot behaves similarly to the responsivity behaviour, the maximum value of detectivity was also determined at a wavelength of 560 nm, as shown in Table 4. The detectivity depends strongly on the responsivity according to the following equation:^19^18
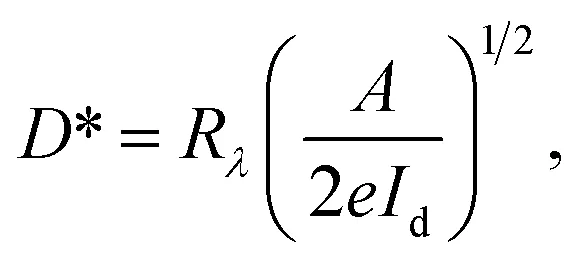
19
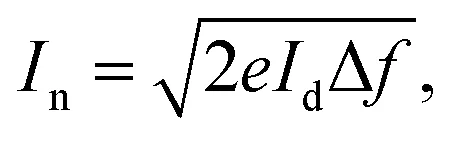
where *I*_n_ is the noise current, *I*_d_ is the dark current, *A* is the active area of window of the photodetector, *e* is the electron charge, and Δ*f* is the frequency bandwidth. The quantitative interpretation of the detection spectrum shows an improvement of up to 90.5% in detection in the visible region, specifically at a wavelength of 560 nm, as a standard definition.20
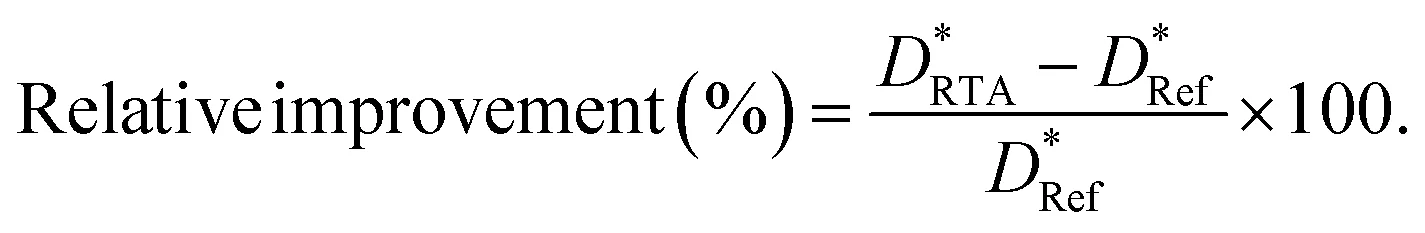


**Table 4:** Summary of the performance parameters (*R*_*λ*_), (EQE), and (D*) of the VN/PSi/*n*-Si photodetector

Figure of merit VN/PSi/*n*-Si	Peak wavelength (nm)				Peak wavelength (nm)		
	480	560	920		480	560	920
*R*_*λ*_ (A W^−1^)	2	2.69	0.32	After RTA	3.05	3.76	0.47
EQE	540%	590.5%	40%		800%	832%	60%
D* (Jones)	1.7 ×10^12^	2.7 ×10^12^	0.2 ×10^12^		3 ×10^12^	3.9 ×10^12^	0.5 ×10^12^

This result confirms the effectiveness of the RTA, as the detector's fundamental characteristics remain unchanged, and only the specific detectivity value is improved. This is further confirmed by the detection ability of the photodetector before RTA, as shown in Fig. 8(c).

The comparison of the figures of merit of the VN/Si photodetector after RTA with some heterojunction photodetectors annealed at various temperatures prepared using different methods is shown in Table 5.

**Table 5:** Comparison of the main parameters of the annealed VN/Si photodetector with those of the other photodetectors

Photodetector type	Annealing temperature (°C)	Photoresponse performance	Major structural effect	Ref.
VO_*x*_/p-Si	300–500	Responsivity at −5 V and under the illumination of IR (1.6 A W^−1^), red (5.6 A W^−1^), green (8.5 A W^−1^), and blue LEDs (3.82 A W^−1^)	Improved crystallinity and reduced defects	42
ZnO/Si	400–800	Responsivity up to 1.77 A W^−1^, detectivity ∼10^11^ Jones, broadband response (254–980 nm)	Enhanced crystallinity and carrier mobility	43
Indium tungsten oxide (IWO)/silicon	350	Responsivity = 3.32 A W^−1^, detectivity = 5.8 × 10^11^ Jones, response time ≈ 1 ms	Reduced defect density and improved interface	44
VN/PSi	RT	Responsivity = 3.7 A W^−1^ detectivity = 6.5 × 10^11^ Jones, EQE = 103% at 500 nm, rise time = 0.18 s, fall time = 0.3 s	VN films have a cubic structure (8 nm crystallite size, 2.98 eV bandgap) with nanoparticles distributed and embedded inside the porous silicon matrix	19
VN/PSi	Rapid thermal annealing	Responsivity up to 3.76 A W^−1^, detectivity = 2.3 to 3.9 × 10^12^ Jones, and EQE increases from 590.5% to 832% at 560 nm	Improved crystallinity, reduced lattice defects, and improved VN/PSi interface	This work

## 4 Conclusion

The effect of rapid thermal annealing on the structural and optical properties of vanadium nitride (VN) thin films was investigated. XRD studies confirmed an improvement in the crystallinity of the film after RTA. The optical energy gap of the film decreased from 3.63 to 3.58 eV after RTA. Annealing the VN films improved the performance of the VN/Si heterojunction photodetector, as evidenced by a decrease in ideality factor and an increase in barrier height after RTA. The photoresponse of the VN/PSi/*n*-Si heterojunction photodetector was significantly enhanced after RTA. The responsivity increased from 2.69 to 3.76 A W^−1^ at 560 nm. A shift in the peak response was also observed after annealing. The specific detectivity and external quantum efficiency improved significantly after RTA, indicating that the post-RTA process enables the photodetector to detect weak optical signals. Despite the promising improvements achieved by RTA, we suggest that further studies are needed to assess the long-term stability and durability of the VN/PSi/*n*-Si photodetector. Future work may focus on RTA scalability, reproducibility, and fabrication optimisation to enhance device performance and reliability.

## Conflicts of interest

The authors declare that they have no known competing financial interests or personal relationships that could have appeared to influence the work reported in this paper.

## Data Availability

The datasets generated and/or analyzed during the current study are available from the corresponding author (R. A. Ismail) on reasonable request.
